# Retrench or Bankrupt

**Published:** 1892-07

**Authors:** H. A. West

**Affiliations:** Secretary, Galveston


For Daniel's Texas Medical Journal.

### "RETRENCH OR BANKRUPT."

### An Open Letter to the Members of the Texas State Medical Association.

H. A. WEST, M. D., SECRETARY, GALVESTON.

An editorial with the above impressive caption appears in DANIEL'S TEXAS MEDICAL JOURNAL of May. Were it not for the fact that the unwary and uninformed might be misled by the apparent imposing array of facts and figures therein contained, I should not trouble myself to make any reply; but solely for the comfort and consideration of those who might not investigate for themselves, I beg leave to present the other side.

There is an old saying, "Figures never lie." If we are to understand lying to be a perversion of the truth, or such a presentation of partial truths as to convey an incorrect impression, then I think I shall be able to show that the figures above mentioned afford an illustration of the fact that figures do lie, and, sometimes egregiously. Let us look at the figures in detail. Dr. Daniel says, "The treasurer's report shows up to the Tyler meeting a total on hand of $2325.00. Under the new order of things all of this vast sum was paid out (to wit, $2190) except $130.00—and for what? The items of disbursement were the same each year, except $175.00 paid the stenographer for work." Looking at the treasurer's report at Tyler, which Dr. Daniel heard read, we find that of this "vast sum" the following items —April 30, by cash paid F. E. Daniel, Secretary's salary, $200; April 30, by cash paid Publishing Committee (Daniel) $300. Now, Dr. Daniel must have known, for he received the money and was present when Dr. Larendon made his report, that of this "vast sum," which he gives the new administration credit of expending, that he (Daniel) pocketed $500.00. Now don't such figures lie? As to the item of $175.00 paid to the stenographer, who made this contract? Dr. Daniel! Whereas I made a contract with the same stenographer to do the same work for $50.00 at the Tyler meeting. Does not Dr. Daniel attempt in his array of figures to make me responsible for this extravagant item, when he knows perfectly well that he made the contract? What about these figures telling the truth? As to the cost of the Transactions, let us see what story these figures tell; but before doing so, I will state a little unwritten history. I had hardly gotten the secretary's chair warm before a letter was received from Dr. Daniel, enclosing one from Von Boeckmann, urging me to give the latter a chance to print [bid on.—Ed.] the Transactions. Upon my reply that I would be compelled to get the work done in Galveston as I could not do it in Austin, Dr. Daniel kindly wrote that he would read and correct proof for me. Here was a cool proposition; I was to play the part of a figure-head. As I had every reason to believe that the Association had elected me because they wanted a change, and as the part of a figure-head is one I never play, and as I proposed to get up a different sort of a book from that gotten out by Dr. Daniel and Von Boeckmann, I promptly replied that I was Secretary and proposed to fill that officer's place. When it came to getting a bid from the printers I found that Clark & Courts had no competitors here, so there was nothing left for me to do except give them the prices which had formerly been paid, and exact a promise that they would do the work as reasonably as possible. As to the precedent of having the work done at Fort Worth when the Secretary lived in Austin, this is a mere quibble; as Drs. Daniel and Broiles, of the committee, lived at Fort Worth in 1884, and I suppose superintended the printing. Concerning the cost of printing the Transactions, certainly it was greater. We got a better book and we paid more for it. As I understand it, we pay for what we get in this world. If we want to compare prices the articles must have the same value. It is unfair to compare the price of a fine hat with a cheap one, or a book wretchedly printed on poor paper and badly bound with one where the workmanship and material is in every way superior. It is unfair to estimate the comparative cost as to the price per page, for everybody knows that printers charge for the numbers of ems; one page may actually contain twice as much matter as another, and would, of course, cost proportionately more. To illustrate—in the Transactions of 1889 the roll of members occupies nineteen pages, in 1891 nine pages;but there were a few more members in 1889. Let us take another illustration. The code of ethics in 1889 takes seventeen pages, in 1891 twelve pages. So any figures, which go to show the comparative cost of the two works according to the price per page, and leaves out of view the comparative amount of matter contained upon the pages, convey an incorrect impression—in other words, such figures lie.

Well, let us compare the books. Take Austin 1890–91 and Galveston 1891–92. Dr. Daniel says he can "see no improvement." "There are none so blind as those who won't see," but a blind man could tell the difference between the two books. He could feel that the Galveston book was printed on better paper and that it was better put together. Then, if he could see even a little bit, he would observe that in the general make-up, execution and arrangement, that the Galveston book was infinitely superior. But let us examine the two books in detail and see if any improvements can be pointed out. Take the book of 1890; it has no table of contents; the book of 1891 has. The book 1890 is printed in the same sized type. Look at the Galveston book and see how it is improved by printing the discussions, etc., in finer type. If any discussions are printed in the Austin book I can't find them. Is it no improvement to have discussions immediately follow the subject? The roll of members in the Austin book was so imperfect and so full of errors that it required the very hardest work and consumed weeks of time to correct it. Is it no improvement to have a fairly accurate list of members? The book of 1890 has no alphabetical index; it has a thing labeled *index* in the back part, but compare it with the index in the Galveston book and one can see it is no index at all. But what is the use of proceeding in this line? The truth is, that the Transactions of the Texas State Medical Association, as published under the auspices of Daniel and Von Boeckmann have made a more wretched appearance than that from Maine to California. And the Association, I take it, was perfectly willing to pay a little more to avoid the disgrace of getting out the worst looking volume of Transactions of any State or Territory in the Union. But what is the actual difference in the cost of the two books? I have shown that it was unfair to compare as to the cost per page, because the pages do not contain the same amount of matter. It is unfair to add the stenographers bill to the cost of the Transactions; and even were it fair, Dr. Daniel, having made the contract, is responsible. The simplest way of getting at the difference in the cost is to compare the cost per volume. We will assume that the book of 1890 and 1891 have the same amount of matter, for though there are thirty-five pages more in 1890,—still, if measured up it would be found that the matter is about equal, —the cost per volume in 1890 was .85 per volume; in 1891, $1.20. [600 copies cost, exclusive of postage, $732, or $1.22 per copy. Do figures lie?—Ed.] In other words, the Association paid .35 [37c., or over 43 per cent.--Ed.] more for a very much better book, and according to my belief they were perfectly willing to do so.

As to the bugbear of bankruptcy, Dr. Daniel need not vex his righteous and economical soul. The money to pay for the coming volume is nearly, if not entirely, in hand. Retrench, yes ! but not in the direction of printing a miserably, cheap, flimsy, wretched-looking volume of Transactions. We look for the gist of a woman's letter in the postscript; so we look to the closing sentence of Dr. Daniel's editorial to find the "milk in the cocoanut." Here it is—"Would it not be well to restore the old Publishing Committee?"

Is Dr. Daniel actuated by a sincere desire to advance the interests of the Association? I fear not. It looks to us as if he is trying to frighten the Association with the idea that it is going to the "demnition bow vows" financially, and that the only salvation is to restore the old Publication Committee. Oh, Daniel, thy name is Modesty.

It has not been my intention in this letter to impute to Dr. Daniel any intentional misrepresentation, but his editorial is an illustration of how a man's views and statements may be biased by his feelings. The trouble with the Doctor is that he imagines that he is the only man in the State competent to act as Chairman of the Publishing Committee. It is partly with the view of dispelling that illusion that I have written to this length.

